# Causes of perinatal mortality and associated maternal complications in a South African province: challenges in predicting poor outcomes

**DOI:** 10.1186/s12884-015-0472-9

**Published:** 2015-02-15

**Authors:** Emma R Allanson, Mari Muller, Robert C Pattinson

**Affiliations:** School of Women and Infants Health, Faculty of Medicine, Dentistry and Health Sciences, University of Western Australia, Perth, Australia; Mpumalanga Department of Health, PPIP Coordinator, Mpumalanga, South Africa; South African Medical Research Council, Maternal and Infant Health Care Strategies Unit, Pretoria, South Africa; Department of Obstetrics and Gynaecology, University of Pretoria, Pretoria, South Africa

**Keywords:** Perinatal mortality, Maternal complication, Growth restriction, Hypertension, Intrapartum care

## Abstract

**Background:**

Reviews of perinatal deaths are mostly facility based. Given the number of women who, globally, deliver outside of facilities, this data may be biased against total population data. We aimed to analyse population based perinatal mortality data from a LMIC setting (Mpumalanga, South Africa) to determine the causes of perinatal death and the rate of maternal complications in the setting of a perinatal death.

**Methods:**

A secondary analysis of the South African Perinatal Problems Identification Program (PPIP) database for the Province of Mpumalanga was undertaken for the period October 2013 to January 2014, inclusive. Data on each individual late perinatal death was reviewed. We examined the frequencies of maternal and fetal or neonatal characteristics in late fetal deaths and analysed the relationships between maternal condition and fetal and/or neonatal outcomes. IBM SPSS Statistics 22.0 was used for data analysis.

**Results:**

There were 23503 births and 687 late perinatal deaths (stillbirths of ≥ 1000gr or ≥ 28 weeks gestation and early neonatal deaths up to day 7 of neonatal life) in the study period. The rate of maternal complication in macerated stillbirths, fresh stillbirths and early neonatal deaths was 50.4%, 50.7% and 25.8% respectively. Mothers in the other late perinatal deaths were healthy. Maternal hypertension and obstetric haemorrhage were more likely in stillbirths (p = <0.01 for both conditions), whereas ENNDs were more likely to have a healthy mother (p < 0.01). The main causes of neonatal death were related to immaturity (48.7%) and hypoxia (40.6%). 173 (25.2%) of all late perinatal deaths had a birth weight less than the 10^th^ centile for gestational age.

**Conclusion:**

A significant proportion of women have no recognisable obstetric or medical condition at the time of a late perinatal death; we may be limited in our ability to predict poor perinatal outcome if emphasis is put on detecting maternal complications prior to a perinatal death. Intrapartum care and hypertensive disease remain high priority areas for addressing perinatal mortality. Consideration needs to be given to novel ways of detecting growth restriction in a LMIC setting.

## Background

Perinatal mortality remains globally unacceptably high with up to three million stillbirths and three million neonatal deaths every year [1,2]. Achievement of Millennium Development Goals (MDG) 4 and 5 requires a focus on antenatal, intrapartum and postpartum perinatal and maternal care [3]. These goals are linked because maternal and perinatal outcomes are inherently linked, and programs addressing improving the care of one often has impact on the outcomes the other, particularly centred around management of hypertension and intrapartum care [4-6].

Accurate population data is necessary to identifying the causes of perinatal mortality and this doesn’t exist in much of the global obstetric population [7,8]. We must ensure we have accurate population assessments of mortality such that we can target current interventions appropriately and plan for future programs efficiently. Pre-term birth, infection, hypertensive disease and intrapartum asphyxia are frequently cited as the most common contributors to perinatal mortality in low and middle income countries (LMICs) [2,4,9,10]. As 43% of deliveries in the least developed countries and only 68% of all deliveries globally occur in institutions [11] (and so facility based data on perinatal mortality is not necessarily reflective of the baseline obstetric population) it may be that these common causes are weighted differently in the total obstetric population. More than 90% of women in the province of Mpumalanga, South Africa, give birth in a health care facility [12], and so we have a unique opportunity to assess perinatal mortality in a population that is reflective of the total obstetric population.

The Perinatal Problem Identification Program (PPIP) in South Africa is a software based quality of care audit system which allows users to capture perinatal deaths as well as potentially modifiable factors in perinatal mortality. PPIP version 3 is currently in use and, in addition to the perinatal death data, there is a requirement that a maternal condition (either that the mother was healthy or had a recognised medical or obstetric pathology at the time of perinatal death) is recorded in every perinatal death.

We analysed data from the Mpumalanga province, South Africa, to outline the causes of late stillbirth and early neonatal death, and the associated maternal complications in these deaths in a representative obstetric population in an LMIC setting.

## Methods

Mpumalanga is the second smallest province in South Africa, covering an area of 76495 square kilometres, with a population of 4039939 as at the last census (2011) [13]. All public obstetric facilities in the province use the PPIP version 3 system. Detailed individual data is entered on each death; births are recorded as amalgamated data. We reviewed data from the PPIP database for the province for the period 1^st^ October 2013 to 31^st^ January 2014, inclusive. This time period represented the first four months of the use of version 3 of PPIP in the province and the compulsory capturing of the maternal condition at the time of perinatal death.

We extracted detailed data on the late fetal deaths (babies weighing 1000gr or ≥ 28 weeks gestation) and early neonatal deaths (deaths up to 7 days of neonatal life). In the time period reviewed there were 23503 births and 687 late fetal and neonatal deaths. Each individual death form was reviewed and the following data was extracted: maternal age, parity, syphilis and HIV status, gestational age, certainty or uncertainty of gestation, condition of the new-born (born alive and early neonatal death (ENND), fresh stillborn, macerated stillborn), primary obstetric cause of death (all cases) and primary neonatal cause of death (ENNDs).

The clinical team at each site perform a death review in the immediate period after a perinatal death has occurred and determine by a consensus decision the primary obstetric cause of death, the primary neonatal cause of death and the maternal condition at the time of perinatal death. The primary obstetric cause of death is defined by the PPIP technical team as the main obstetric event or pregnancy occurrence which was integral in the pathway to perinatal death. As we are a low resource setting, this decision comes from case review and verbal autopsy and rarely from placental histology or fetal/neonatal autopsy. The primary obstetric causes of death are grouped under the headings of spontaneous preterm labour, infections, antepartum haemorrhage, intrauterine growth retardation, hypertensive disorders, fetal abnormality, trauma, intrapartum asphyxia, maternal disease, miscellaneous (rhesus isoimmunisation, twin-to-twin transfusion, extra-uterine pregnancy and other cause of death not described), intrauterine death and no obstetric cause. The review also identifies the primary neonatal cause of death in the same way; these are categorised under immaturity related, hypoxia, infection, congenital abnormalities, trauma, miscellaneous and unknown cause of death. The maternal condition is defined as either healthy or the occurrence of a recognised medical or obstetric complication, categorised as coincidental conditions, medical and surgical disorders, non-pregnancy related infections, extrauterine pregnancy, pregnancy-related sepsis, obstetric haemorrhage, hypertension, anaesthetic complications, embolism, and acute collapse (cause unknown).

Where there was a fresh stillborn as a result of labour related intrapartum asphyxia, meconium aspiration or traumatic delivery or an ENND as the result of hypoxic ischaemic encephalopathy (HIE) or meconium aspiration syndrome (MAS), we reviewed the avoidable factors identified in each death for factors that would indicate that the intrapartum event was a result of poor intrapartum care. Avoidable factors are identified as a part of the death review by the on site team in each individual case. The PPIP program lists 69 possible avoidable factors that staff may identify in a case. The factors we examined were fetal distress not detected intrapartum, management of the second stage prolonged or inappropriately managed, delay in medical personnel calling for assistance, no or inadequate response to maternal hypertension, delay in accessing anaesthetic, delay in referring patient for secondary or tertiary care, under or over-estimation of fetal size by medical personnel and inadequate neonatal resuscitation.

In order to examine the potential contribution of growth restriction to late perinatal death, we used Theron charts (South African specific plots) for birth weight for gestation and defined growth restriction as country specific weight less than the 10^th^ centile for gestational age [14,15].

The data is collected and entered on site by trained data collectors, who have all been trained by a single person. That same person undertakes a constant visiting cycle of the health services. At these visits the patient files for the deaths are reviewed against the data entered in order to quality control, teach and provide feedback to the staff. This process is particularly focused on the unexplained stillbirths, in order to ensure identification of contributing factors and potential causes in cases where staff has not identified these. This case review is done not only with the data collectors but with the clinical team, a process which aids in the closing of the audit loop.

We examined the frequencies of maternal and fetal or neonatal characteristics in late fetal deaths and analysed the relationship between maternal condition and fetal or neonatal outcomes. IBM SPSS Statistics 22.0 was used for data analysis. Frequency distributions were performed for outcome summaries. Fisher’s exact test on bivariate correlation was used to interrogate the relationship between maternal conditions and obstetric causes of death and the timing of late perinatal death. Analysis outcomes are summarised using risk ratios and 95% confidence intervals. All tests were two tailed and p-values <0.05 were considered significant.

The PPIP program has ethical approval from the University of Pretoria. The data is collected with permission from the South African Department of Health. This secondary analysis was approved by the PPIP technical task team. The Mpumalanga Department of Health granted permission for analysis and publication of the Province’s PPIP data.

## Results

Two hundred and sixty six macerated stillbirths (38.7% of late perinatal deaths), 150 (21.8%) fresh stillbirths and 271 (39.4%) early neonatal deaths occurred in the study period. The fetal/neonatal and maternal characteristics are presented in Table 1. Four hundred and twenty 21 (61.3%) of the deaths were born alive or were fresh stillbirths and 462 (67.2%) of deaths had a certain gestation.

**Table 1:** **Fetal/neonatal and maternal characteristics of macerated and fresh stillbirths and early neonatal deaths**

	**Macerated stillbirth**	**Fresh stillbirth**	**Early neonatal death**
	**(n = 266)**	**(n = 150)**	**(n = 271)**
	**n(%)**	**n(%)**	**n(%)**
**Maternal age**			
<20	44(16.5)	25(16.7)	48(17.7)
20-34	175(65.8)	103(68.7)	184( 67.9)
≥35	45(16.9)	22(14.7)	33(12.2)
Missing	2(0.8)		6(2.2)
**Parity**			
0	124(46.6)	64(42.7)	104(38.4)
1-2	99(37.2)	62(41.3)	118(43.5)
>2	39(14.7)	20(13.3)	31(11.4)
Missing	4(1.5)	4(2.7)	18(6.6)
**Positive syphilis status**	8(3)	0	3(1.1)
**Positive HIV status**	88(33.1)	36(24)	111(41)
**Gestational age**			
>42	0	1(0.7)	0
37-42	83(31.2)	64(42.7)	92(33.9)
32-36	88(33.1)	46(30.7)	56(20.7)
28-31	79(29.7)	31(20.7)	52(19.2)
<28	12(4.5)	4(2.7)	47(17.3)
Unknown	4(1.5)	4(2.7)	
**Certain gestation**	168(63.2)	110(73.3)	184(67.9)
**Birth weight**			
<1500	81(30.5)	23(15.3)	121(44.6)
1500-2499	104(39.1)	46(30.7)	55(20.3)
2500-3999	74(27.8)	76(50.7)	93(34.3)
>4000	7(2.6)	5(3.3)	2(0.7)
**Growth <10** ^**th**^ **centile**	79 (29.7)	24(16)	70(25.8)

Four hundred and seven (59%) maternal conditions in late perinatal death were recorded as healthy. One hundred and thirty two (49.6%) macerated stillbirths, 74 (49.3%) fresh stillbirths and 201 (74.2%) early neonatal deaths had a healthy mother. A healthy mother was more likely in an early neonatal death compared with macerated stillbirth (RR 1.8 (1.4-2.2), p = <0.01), or fresh stillbirth (RR 1.5 (1.3-1.8), p < 0.001). Where there was a maternal condition, the most common were hypertension and obstetric haemorrhage. Both were more likely in stillbirth than early neonatal death (Table 2).

**Table 2:** **Comparison of maternal condition in late stillbirth and early neonatal deaths**

	**Late stillbirths**	**Early neonatal deaths**	**X** ^**2**^
	**(n = 416)**	**(n = 271)**	
	**n (%)**	**n (%)**	
**Healthy mother**	**206 (49.5)**	**201 (74.2%)**	**<0.001**
**Coincidental conditions**			
Assault	2 (0.5)	0	
Herbal medicine	10 (2.4)	5 (1.8)	
MVA	2 (0.5)	0	
Other coincidental conditions	1 (0.2)	0	
**Medical and surgical disorders**			
Maternal medical condition			
Auto-immune	0 (0)	1 (0.4)	
Endocrine disease	5 (1.2)	1 (0.4)	
Gastroenteritis	1 (0.2)	0	
Genito-urinary disease	5 (1.2)	1 (0.4)	
GIT disease	0	1 (0.4)	
Haematological disease	1 (0.2)	0	
Urinary tract infection	7 (1.7)	3 (1.1)	
Other medical and surgical disorders	1 (0.2)	1 (0.4)	
**Non-pregnancy related infections**			
Complications of ARV therapy	1 (0.2)	1 (0.4)	
TB	3 (0.7)	0	
Varicella	1 (0.2)	0	
Wasting syndrome	2 (0.5)	1 (0.4)	
**Pregnancy related sepsis**			
Chorioamnionitis	3 (0.7)	3 (1.1)	
**Obstetric haemorrhage**	**64 (15.4)**	**20 (7.4)**	**0.002**
Abruption	52 (12.5)	9 (3.3)	<0.001
Placenta praevia	6 (1.4)	3 (1.1)	
Other APH not specified	4 (1)	8 (3)	0.072
Ruptured uterus	2 (0.5)	0	
**Hypertension**	**96 (23.1)**	**23 (8.5)**	**<0.01**
Chronic HTN	3 (0.7)	1 (0.4)	
PIH	12 (2.9)	2 (0.7)	0.057
Pre-eclampsia	74 (17.8)	15 (5.5)	<0.01
Eclampsia	6 (1.4)	5 (1.8)	
HELLP	1 (0.2)	0	

The obstetric causes of perinatal death are outlined in Table 3. In addition to unexplained intrauterine deaths, intrapartum asphyxia, hypertensive disorder and spontaneous preterm labour remain the most common obstetric causes of death. In the 271 early neonatal deaths, the main final neonatal causes of death were immaturity related and hypoxia (Table 4). The hypoxia deaths were as a result of both HIE (69) and MAS (41). The average birth weight of these babies was 2884gr, with an average gestational age of 37.3 completed weeks. 81.2% (n = 56) of the HIE cases had a healthy mother, as did 82.9% (n = 34) of the MAS cases. By comparison, the neonates that died of HMD (n = 55) or extreme immaturity (n = 71) had an average birth weight of 1060gr and an average gestational age of 28.8 completed weeks.

**Table 3:** **Obstetric causes of death in late stillbirth and early neonatal death**

	**Late stillbirth**	**Early neonatal**	**X2**
	**(n = 416)**	**(n = 271)**	
	**n(%)**	**n(%)**	
Spontaneous preterm labour	5(1.2)	104(38.4)	<0.001
Infections	7(1.7)	1(0.4)	0.156
Antepartum haemorrhage	68(16.3)	20(7.4)	0.001
Intrauterine growth restriction	4(1)	0	
Hypertensive disorders	98(23.6)	22(8.1)	<0.001
Fetal abnormality	12(2.9)	12(4.4)	0.294
Trauma	3(0.7)		
Intrapartum asphyxia	66(15.9)	106(39.1)	<0.001
Maternal disease	6(1.4)	1(0.4)	0.255
Miscellaneous	3(0.7)		
Unexplained intrauterine death	144(34.6)		

Note: 5 late stillbirths had no obstetric cause of death identified.

**Table 4:** **Causes of neonatal death**

**Neonatal cause of death**	**n (%)**
Immaturity related	132 (48.7)
Hypoxia	110 (40.6)
Infection	8 (3)
Congenital abnormalities	15 (5.5)
Trauma	0
Miscellaneous*	5 (1.8)
Unknown	1 (0.4)

* Miscellaneous (Haemorrhagic disease of the newborn, other cause of death, sudden infant death syndrome).

There was a combined total of 147 FSB as a result of labour related intrapartum asphyxia, meconium aspiration or traumatic delivery and ENND as the result of hypoxic ischaemic encephalopathy or meconium aspiration syndrome. Eighty nine (60.5%) had one of the avoidable factors related to poor intrapartum care identified as contributing to the outcome.

Growth less than the 10^th^ centile was found in 173(25.2%) of all late perinatal deaths and 106 of these (61.3%) cases also had a healthy mother. One hundred and one of the 462 deaths with certain gestation had a birth weight less than the 10^th^ centile for gestation, representing 21.9% of these deaths. The average weight of growth restricted cases was 1484gr and 122 (70.5%) were 32 weeks gestation or greater.

## Discussion

The prevention and management of intrapartum asphyxia, hypertensive disorders and spontaneous preterm labour remain clear priority areas for reducing perinatal mortality. The rate of intrapartum asphxyia in our data is not disimilar to studies in similar settings [16,17], however the structure of PPIP allows us to consider the role of avoidable factors in these deaths. That the majority of FSB and ENND following an intrapartum event had an avoidable factor relating to intrapartum care and that ENNDs were more likely to have a healthy mother is very concerning. Given that we cannot predict these poor outcomes antenatally by assessment of the mother, and we are contributing to the outcome through poor intrapartum care, it is critical that care-providers of all women, regardless of antenatal risk, are trained in the management of obstetric emergencies and intrapartum care.

Where there is a recognised maternal condition, obstetric haemorrhage (particularly abruption) and hypertension (particularly pre-eclampsia) are more likely to contribute to late stillbirths than ENNDs. This seems to be the group where we have some ability to predict poor perinatal outcomes. In giving consideration to the management of hypertension, one can also consider that addressing this with the aim of reducing perinatal mortality may also impact maternal health outcomes; as shown in the WHO Multicountry Survey on Maternal and Newborn Heath, women with pre-eclampsia and eclampsia have significantly increased rates of both near miss and death compared to those without the disease [18].

The rate of potential growth restriction in this population data was surprising and represents an area of obstetric care in LMICs to which attention is required. This is particularly pertinent given the deaths with growth restriction were largely of a large enough weight and old enough gestation that one would expect a reasonable chance of survival if born alive [19,20]. The challenges of detecting growth restriction in LMICs centre on the frequent lack of early accurate dating of pregnancy and the limitations of examination to detect growth restriction in combination with the lack of resources, namely ultrasound, to support or refute any clinical suspicion. There is no clear evidence that symphysis fundal height is able to predict growth restriction [21], however this is currently the sum total of our toolkit for screening. There are obvious limitations to using the definition of the 10^th^ centile based on a single plot of birth weight and gestational age; it is impossible to distinguish the constitutionally small fetus or the fetus above the 10^th^ centile that has had a significant drop in weight velocity [22]. However we cannot dismiss such a finding out of hand and consideration must be given to an increased focused on growth restriction in LMICs.

Given that nearly two thirds of the deaths where growth restriction was found also had a healthy mother, consideration also needs to be given to the ability to detect growth restriction in a well and, quite likely, otherwise low risk population in LMICs. The ideal early dating and other antenatal imaging require human and physical resources which are outside of the capability of our setting. One must reflect on other ways of detecting growth restriction within the limitations of our resources. Indicated umbilical artery Doppler studies in the developing world have been shown to be associated with increased perinatal mortality when absent or reversed [23] but the challenge for us is the significant number of perinatal deaths in this population where growth restriction was associated with a healthy mother. Given this plus the fact that the diagnosis of growth restriction was made on examination of the data after death had occurred, it seems likely that very few of these infants would have qualified for indicated antenatal fetal umbilical artery Doppler screening. A simpler continuous wave Doppler analyser using a PC (Umbiflow) has been shown to be comparable with standard measurements of umbilical artery Doppler flow in high risk populations in South Africa [24] however use of such potentially appropriate technologies has not been tested in the unselected population in a LMIC. It is not clear if this technology has the potential as a screening tool in an unselected population such as we found in the interrogation of these perinatal deaths and further research is required.

Vogel et al. in the WHO Multi-Country Survey [25] found a maternal complication rate of 22.9%, 27.7% and 21.2% in late MSB, late FSB and ENNDs respectively. While our rate of complication in ENNDs was almost identical, the late MSB and FSB in this population had almost double the maternal complication rate (composed almost exclusively of obstetric haemorrhage and hypertension), although still only half of women. The WHOMCS population was drawn from facilities that have reasonable large numbers of deliveries (at least 1000 per year) and the ability to perform caesarean section and so the data may represent more cases with higher clinical acuity. It may be that the women in our population are not being referred to centres similar to this as the stillbirth occurs at a lower referral centre and the patient journey ends there. Our dataset represents total population data (all levels of facilities in a province where >90% of women deliver in a facility) and, given the numbers of babies born globally outside facilities with as many deliveries as in the WHOMCS set [26,27], it is important that we do not lose focus on training all care providers in both the detection of maternal complication and the routine and emergency care of healthy, apparently low risk women who are having poor perinatal outcomes.

### Limitations

This data is retrospective and the events surrounded the maternal and fetal story are only captured after the death has occurred. We have no data on the rates of maternal events, birth weight for gestational age and factors suggesting poor intrapartum care in the cases without a perinatal death. The in depth case review of the unexplained stillbirths by the data trainer could be considered both a strength and a weakness. While we may bias the collection towards finding a maternal condition or an avoidable factor as a consequence of the process of second review, we may also counter the underestimation of maternal condition occurring before a death.

### Strengths

The main strength of this paper is it represents a total obstetric population, with a large number of cases (both births and deaths) in the study period. The requirement that a maternal condition is documented in every case avoids the likely underestimation that comes when perinatal and maternal outcome are not mandated to be interlinked.

## Conclusion

Intrapartum care and management of hypertension and obstetric haemorrhage remain high priority areas for reducing perinatal mortality in a LMIC setting. Consideration needs to be given to novel ways to predict growth restriction in resource limited settings as this may be an underestimated significant contributor to perinatal mortality. A large numbers of perinatal deaths in a LMIC population may be in the context of healthy mothers, limiting our ability to predict poor outcomes by maternal assessment antenatally. Identifying causes and the interplay of maternal and perinatal condition are important in narrowing this focus.

## Abbreviations

END: Early neonatal death
HIE: Hypoxic ischaemic encephalopathy
LMIC: Low and middle income country
LFD: Late fetal death
LND: Late neonatal death
MAS: Meconium aspiration syndrome
MDG: Millennium Development Goals
PNMR: Perinatal mortality rate
PPIP: Perinatal Problems Identification Program
